# Supplementary figures and images for: Development of non-alcoholic steatohepatitis is associated with gut microbiota but not with oxysterol enzymes CH25H, EBI2, or CYP7B1 in mice
Source: BMC Microbiol. 2024 Feb 28;24:69. doi: 10.1186/s12866-024-03195-7 (PMC10900623; doi:10.1186/s12866-024-03195-7)

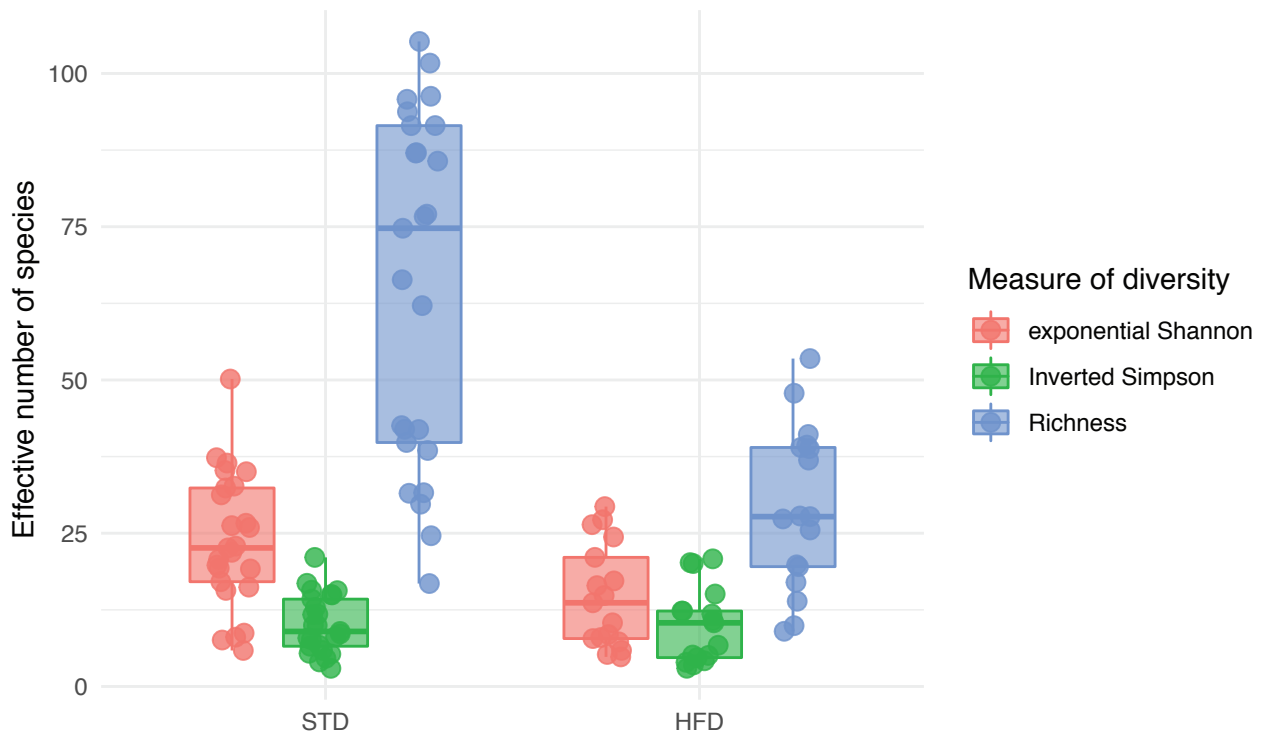

Supplement: Supplementary file 1 — Additional file 1: Fig. S1. Comparison of samples between diet types according to alpha-diversity measures. Comparison of samples from the standard diet and the high-fat diet by diversity indices (species richness, exponential Shannon index, inverse Simpson index) measured by the effective number of species. [file 12866_2024_3195_MOESM1_ESM.pdf]

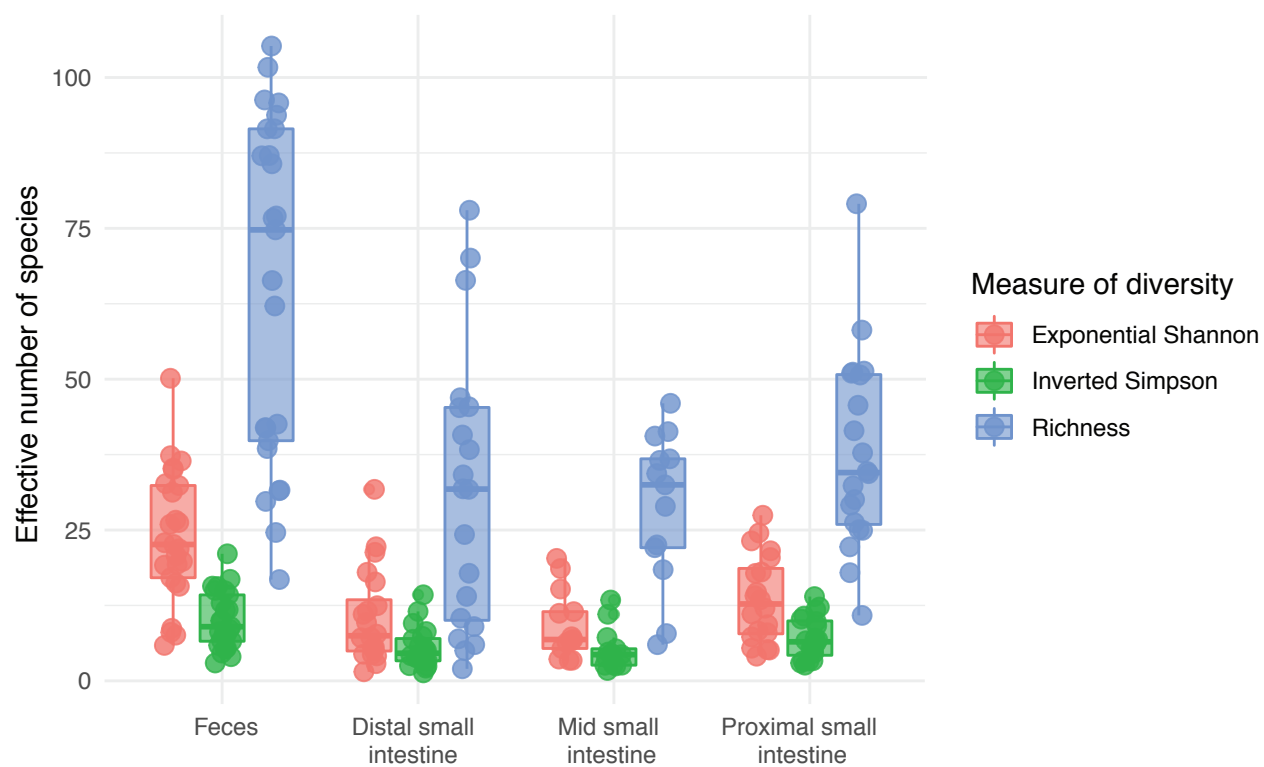

Supplement: Supplementary file 2 — Additional file 2: Fig. S2. Comparison of samples between body sites according to alpha-diversity measures. Comparison of samples from intestinal sampling locations and feces by diversity indices (species richness, exponential Shannon index, inverse Simpson index) is shown, assessed by the effective number of species. [file 12866_2024_3195_MOESM2_ESM.pdf]

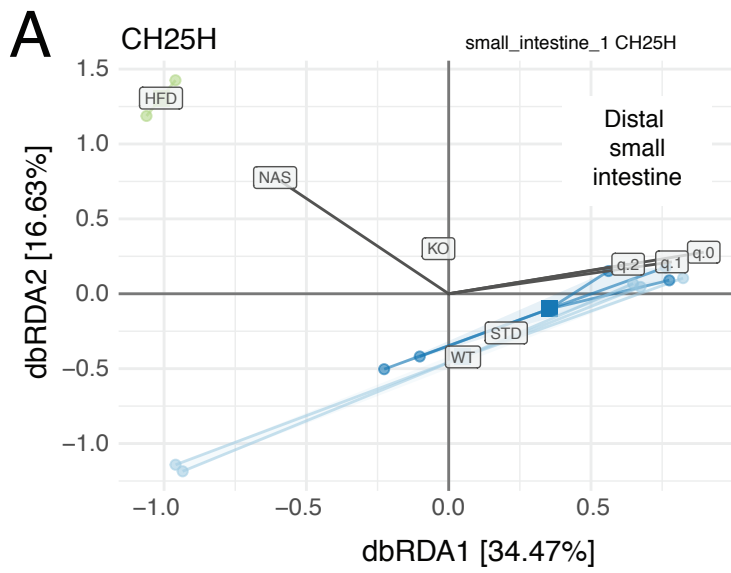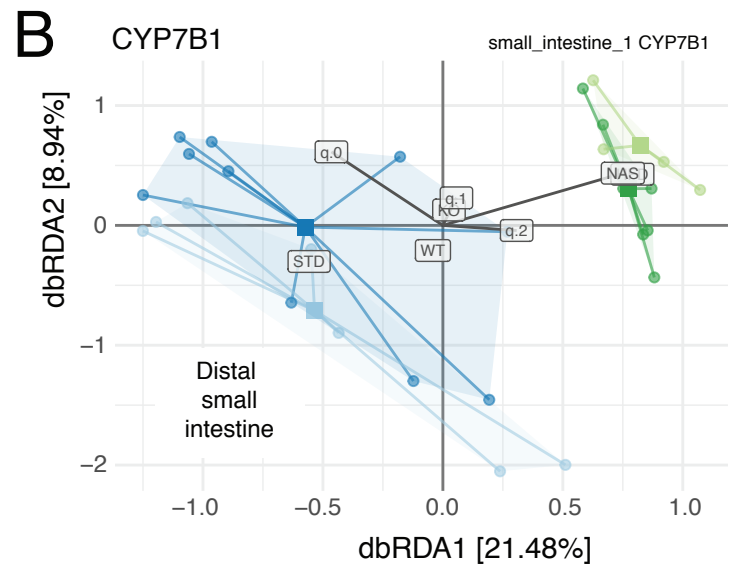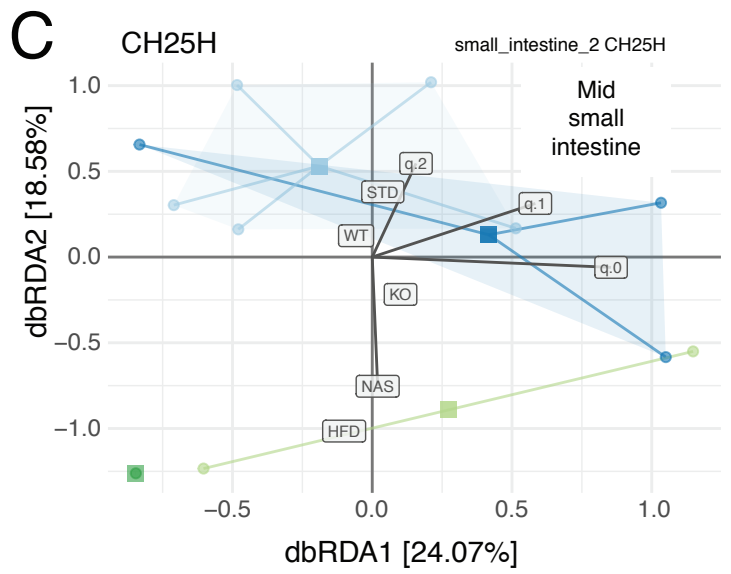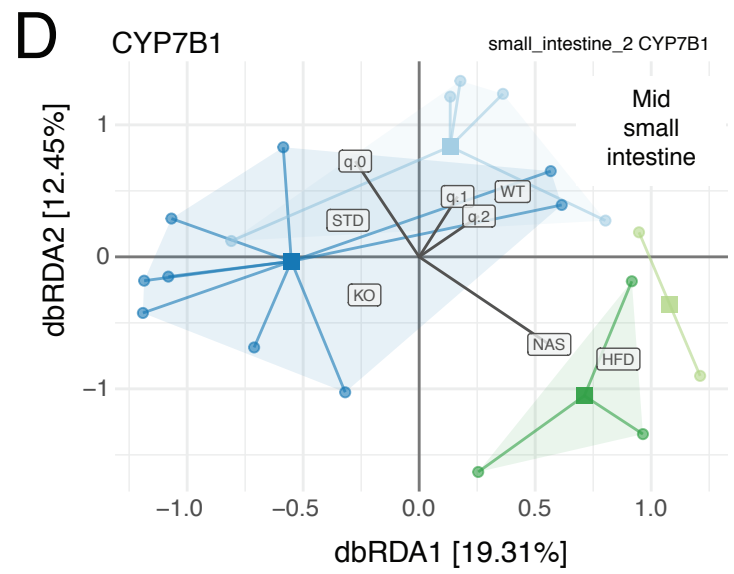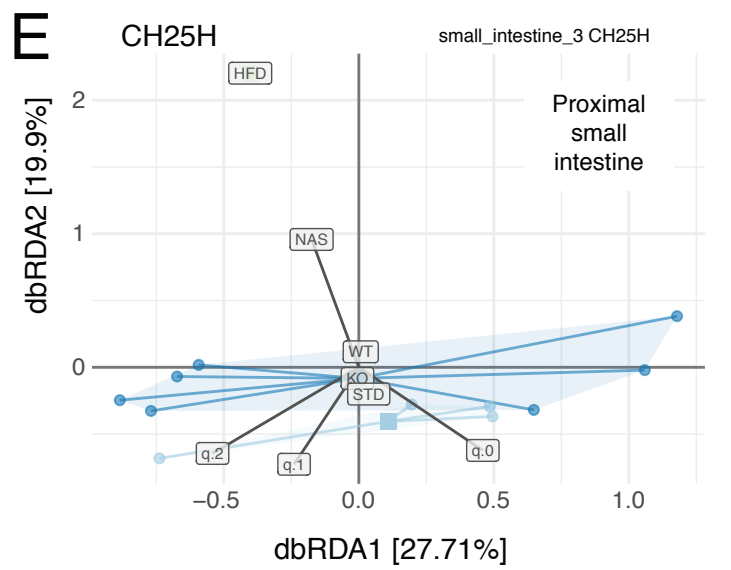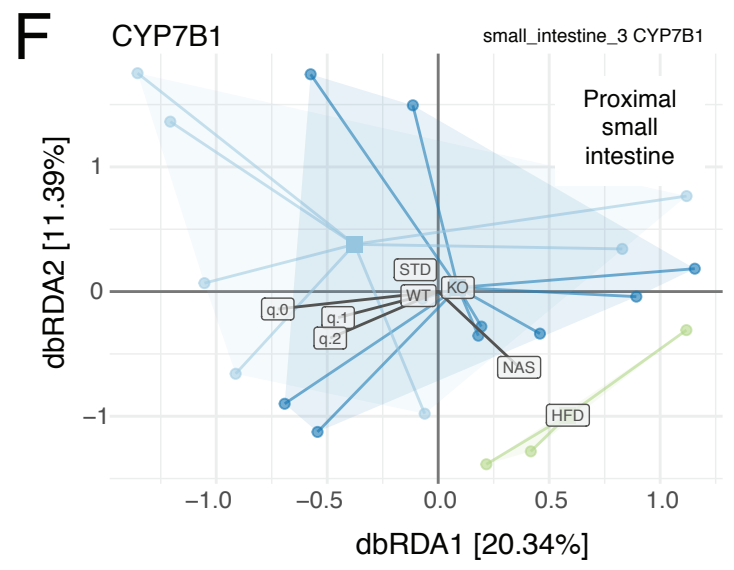

Supplement: Supplementary file 3 — Additional file 3: Fig. S3. Distance-based redundancy analysis of small intestinal microbiota samples according to genotypes with diet, genotype, and NASH status as explanatory variables. Samples are colored according to feeding type (blue: standard diet, green: high-fat diet). A: Distal small intestine, Ch25h-/- (dark/light) versus wildtype (n=7 vs. 5). B: Distal small intestine, Cyp7b1-/- (dark/light) versus wildtype (n=18 vs. 11). C: Mid small intestine, Ch25h-/- (dark/light) versus wildtype (n=4 vs. 7). D: Mid small intestine, Cyp7b1-/- (dark/light) versus wildtype (n=12 vs. 7). E: Proximal small intestine. Ch25h-/- (dark/light) versus wildtype (n=7 vs. 5). F: Proximal small intestine, Cyp7b1-/- (dark/light) versus wildtype (n=10 vs. 10). No analysis of Ebi2-/- was feasible since only in a few small intestinal HFD samples from Ebi2-/- animals (9 out of 33 HFD Ebi2-/- animals) meaningful sequencing results were obtained. [file 12866_2024_3195_MOESM3_ESM.pdf]
